# High-Dose Corticosteroids for a Pregnant Woman Critically Ill With Coronavirus Disease 2019

**DOI:** 10.7759/cureus.17398

**Published:** 2021-08-23

**Authors:** Koichiro Yamamoto, Hideharu Hagiya, Jota Maki, Shuji Okahara, Kou Hasegawa, Fumio Otsuka

**Affiliations:** 1 Department of General Medicine, Okayama University Graduate School of Medicine, Dentistry and Pharmaceutical Sciences, Okayama, JPN; 2 Department of Obstetrics and Gynecology, Okayama University Graduate School of Medicine, Dentistry and Pharmaceutical Sciences, Okayama, JPN; 3 Department of Anesthesiology and Resuscitology, Okayama University Graduate School of Medicine, Dentistry and Pharmaceutical Sciences, Okayama, JPN

**Keywords:** covid-19, cytokine storm, pneumonia, pregnancy, steroids

## Abstract

Pregnancy was reported to be a risk factor for coronavirus disease 2019 (COVID-19), with an increased risk for premature birth. Corticosteroids and remdesivir are used for patients with COVID-19; however, there is no established treatment for these patients. In particular, the effective management of pregnant, critically ill patients with COVID-19 remains unknown. We describe a 34-year-old, critically ill woman at 30 weeks of gestation with COVID-19, who was successfully treated with remdesivir and combined high-dose betamethasone (12 mg/day for two days) and methylprednisolone (125 mg/day for three days) followed by steroid tapering. During treatment, fetal biophysical profile scores on obstetric ultrasound were normal; her pregnancy course progressed well. Since high-dose corticosteroids improve fetal lung maturation and as well as cytokine storm due to COVID-19, this case provides an insight into the management of pregnant COVID-19 patients.

## Introduction

Coronavirus disease 2019 (COVID-19), in which severe acute respiratory syndrome coronavirus 2 (SARS-CoV-2) induces cytokine storm and respiratory failure, has become a huge threat to human lives without the exception of pregnant women [1-3]. COVID-19 severely affects morbidity and mortality; treatment plans have yet to be established, including for pregnant patients [2-5]. Some drugs, such as corticosteroids and remdesivir, are currently used as the standard treatment for COVID-19, however, their effectiveness and safety profile for pregnant COVID-19 patients remains unclear [2-5]. Therefore, the management of pregnant, critically ill patients is considerably challenging. Here, we present a pregnant, critically ill patient with COVID-19, for whom a combination of high-dose corticosteroids, betamethasone and methylprednisolone appeared to be effective.

## Case presentation

A 34-year-old Japanese woman, at 28 weeks and six days of gestation, developed fever and cough. SARS-CoV-2 was detected by loop-mediated isothermal amplification from the saliva sample, and the lineage of the SARS-CoV-2 variant was identified as B.1.1.7 by next-generation sequencing analysis. The patient had a medical history of bronchial asthma without a history of smoking. Since the patient had mild symptoms, initially she was managed symptomatically in outpatient. On the third day of COVID-19 development, she was admitted to the former hospital. On day seven, the patient desaturated to a saturation of percutaneous oxygen (SpO_2_) of 93% and ground-glass opacification in the bilateral lower lobes on chest X-ray appeared. Oxygen supply at 2 L/min through a nasal cannula and administration of prednisolone at 40 mg per day were initiated. However, her respiratory condition progressively deteriorated. The patient was transferred to our tertiary hospital for higher level of care.

**Figure 1:**
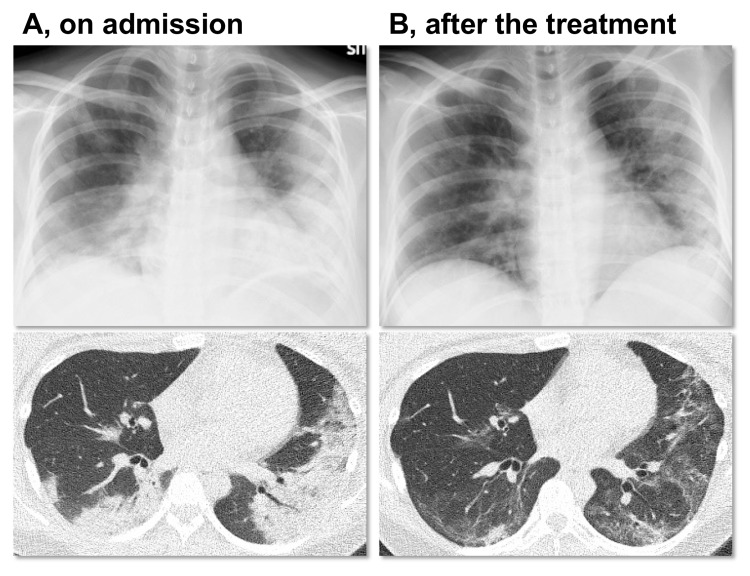
Imaging findings of COVID-19 pneumonia (A) Chest X-ray (upper) and non-contrast computed tomography (CT) (lower) showed multifocal ground-glass opacities with consolidation in the bilateral lungs on admission. (B) Findings of chest X-ray (upper) and non-contrast CT (lower) ameliorated after the treatment of COVID-19 (on day 19).

On admission, her body mass index was 26.7 kg/m^2^. She had a body temperature of 37.0°C, a SpO_2_ of 94% at 4 L/min of oxygen supply through a mask, and a respiratory rate (RR) of 20 /min. Hypotension (92/58 mmHg) and sinus tachycardia (107 /min) were observed as well. No leg edema was observed. Laboratory data showed elevated serum inflammatory markers (C-reactive protein, 6.90 mg/dL; procalcitonin, 1.120 ng/mL). As shown in Table 1, she had slight normocytic anemia and mild liver injury, without specific antibodies for hepatitis B and C viruses. Chest computed tomography (CT) and radiography showed bilateral multifocal ground-glass opacities with consolidation, which were compatible with COVID-19 pneumonia (Figure 1A).

**Table 1:** Laboratory data on admission ALP: alkaline phosphatase; ALT: alanine aminotransferase; AST: aspartate aminotransferase; APTT: activated partial thromboplastin time; BNP: brain natriuretic peptide; ESR: erythrocyte sedimentation rate; γ-GTP: γ-glutamyl transpeptidase; HB: hepatitis B; HCV: hepatitis C virus; IFCC: International Federation of Clinical Chemistry method; LDH: lactate dehydrogenase; MCV: mean corpuscular volume; PT-INR: prothorombin time-international normalized ratio; TSH: thyroid-stimulating hormone.

Hematological data		Units (normal range)
White blood cells	11,030	/μL (3,300-8,600)
Hemoglobin	11.1	g/dL (11.6-14.8)
MCV	93.0	fL (83.6-98.2)
Platelets	197,000	/μL (158,000-348,000)
Biochemical data		Units (normal range)
Blood urea nitrogen	3.9	mg/dL (8.0-20.0)
Creatinine	0.45	mg/dL (0.46-0.79)
Sodium	136	mmoL/L (138-145)
Potassium	3.5	mmoL/L (3.6-4.8)
Chloride	106	mmoL/L (101-108)
Calcium	8.0	mg/dL (8.8-10.1)
Magnesium	1.9	mg/dL (2.0-2.5)
Inorganic phosphate	2.0	mg/dL (2.7-4.6)
AST	83	U/L (13-30)
ALT	32	U/L (7-23)
ALP (IFCC)	200	U/L (38-113)
γ-GTP	65	U/L (9-32)
Total bilirubin	0.53	mg/dL (0.40-1.50)
Creatine kinase	73	U/L (41-153)
Amylase	47	mg/dL (44-132)
Inflammatory markers		Units (normal range)
LDH (IFCC)	412	U/L (124-222)
Ferritin	144.0	ng/mL (6.2-138.0)
C-reactive protein	6.90	mg/dL (<0.15)
Procalcitonin	1.120	ng/mL (<0.050)
ESR	64	mm/h (3-15)
KL-6	536	U/mL (<500)
Coagulation		Units (normal range)
PT-INR	0.87	(≦2.99)
APTT	36.4	sec (26.9-38.1)
Fibrinogen	502	mg/dL (200-400)
D-dimer	1.4	μg/mL (0.0-0.9)
Metabolism and endocrine		Units (normal range)
Total protein	5.7	g/dL (6.6-8.1)
Albumin	2.4	g/dL (4.1-5.1)
Uric acid	4.1	mg/dL (2.6-5.5)
Hemoglobin A1c	5.5	% (4.9-6.0)
TSH	0.11	μU/mL (0.33-4.05)
Free thyroxine	0.84	ng/dL (0.97-1.69)
BNP	30.2	pg/mL (0.0-18.4)
Infection		Units (normal range)
HBs antigen	<0.005 (-)	IU/mL
HBs antibody	<3.0 (-)	mIU/mL (<10.0)
HCV antibody	<1.0 (-)	COI
T-spot	(-)	
β-D-glucan	10 (-)	pg/mL

Her respiratory status rapidly worsened; oxygen supplementation through a high-flow nasal cannula (HFNC), with a fraction of inspired oxygen (FiO_2_) of 0.7 at 40 L/min, was started on day nine in the intensive care unit (ICU). Additionally, she had been perceiving fetal movement. Obstetric ultrasound revealed that the fetal biophysical profile score (BPS) was 10 points; the estimated fetal weight (EFW) was normal (1,700 g (1.2SD)).

**Figure 2:**
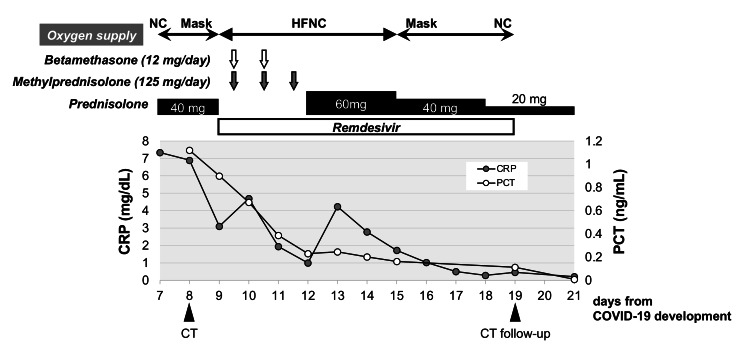
Clinical course The treatment for COVID-19 was started with prednisolone at 40 mg per day, but her respiratory condition deteriorated. Oxygen supply through a high-flow nasal cannula (HFNC) was commenced on the 9th day of the development of COVID-19. In addition to remdesivir administration, a steroid pulse therapy of betamethasone and methylprednisolone with subsequent steroid tapering improved her respiratory condition and serum inflammatory markers (CRP, C-reactive protein; PCT, procalcitonin).

We commenced remdesivir therapy for 10 days and as well as high-dose corticosteroid treatment with 12 mg of betamethasone per day for two days and 125 mg of methylprednisolone per day for three days. These high-dose corticosteroids were initiated to improve fetal lung maturation for the possibility of premature delivery and to suppress a potential cytokine storm caused by COVID-19. Thereafter, her symptoms and serum inflammatory markers improved with subsequent steroid tapering (Figure 2). Oxygen supply through the HFNC was reduced gradually to 0.4 of FiO_2_ and decreased to 5 L/min through a mask on day 15. Findings of COVID-19 pneumonia in chest CT and X-ray improved after treatment (Figure 1B). Corticosteroids were administered for a total of 14 days. In the clinical course, she was administered heparin, with no findings of thrombosis or embolism. After discharge, her EFW (1,910 g (0.7SD)) and BPS (10 points) remained normal on day 25. The present case was planned to be followed until her delivery date.

## Discussion

A living systematic review and meta-analysis revealed that pregnant women with COVID-19 have increased risks for being admitted to the ICU and subsequently receiving invasive respiratory support, while less manifesting symptoms of fever, dyspnea, and myalgia [2]. Infants from mothers with COVID-19 tended to be delivered preterm. In addition, the risks for maternal death and being admitted to the ICU could be increased [2].

To date, several therapeutic options, such as corticosteroids and remdesivir, have been proposed for COVID-19; however, there are no established treatments for COVID-19 [4,5]. A meta-analysis revealed that corticosteroid, especially dexamethasone and not hydrocortisone or methylprednisolone, was associated with an improvement of 28-day all-cause mortality in critically ill COVID-19 patients [6]. In that study, however, doses of corticosteroid were not precisely investigated. According to the preliminary study of the RECOVERY trial, 6 mg of dexamethasone per day for up to 10 days for COVID-19 patients receiving oxygen is widely recommended [1]. In addition, a randomized controlled trial suggested that high-dose methylprednisolone (250 mg per day for three days) could contribute to the clinical improvement and decrease mortality for the early pulmonary phase of severe COVID-19 [7]. Also, an observational comparative study suggested that methylprednisolone pulses (125-250 mg/day for three days) during the second week of COVID-19 decreased the need for intubation or COVID-19-related mortality [8].

In accordance with the Japanese Guide to the treatment of COVID-19, Version 4.2 (https://www.mhlw.go.jp/content/000742297.pdf), our patient was started on treatment with low-dose prednisolone (40 mg per day), not dexamethasone, because of pregnancy. However, she progressed to a critically ill condition. Due to the concern about the possibility of cesarean section, 12 mg of betamethasone per day for two days was administered, which was widely used in Japan to accelerate fetal lung maturation and improve neonatal respiratory distress syndrome, necrotizing enterocolitis, and mortality [9]. Methylprednisolone pulse therapy (125 mg per day for three days) was added to suppress a COVID-19-invoked cytokine storm in the maternal body. On the other hand, prolonged administration of corticosteroids is not recommended because of possible adverse effects on the fetus, such as the development of fetal adrenal insufficiency and impaired childhood neurodevelopment [10,11].

Remdesivir was reported to be associated with an improvement in the 28-day recovery and respiratory support [12]. The antiviral agent is not recommended for routine use in pregnant women; however, since our case was critically ill, its benefits for the reduction of the recovery time were considered [1,12,13]. Adverse events, including liver or renal injury related to remdesivir, were not observed in the clinical course.

## Conclusions

In summary, we reported a case of a critically ill pregnant woman with COVID-19, to whom remdesivir and a combination of high-dose corticosteroids were administered. It is conceivable that short-term use of antenatal high-dose corticosteroids, which could accelerate fetal lung maturation, improved the COVID-19 pneumonia.
